# A Novel Surface Structure Consisting of Contact-active Antibacterial Upper-layer and Antifouling Sub-layer Derived from Gemini Quaternary Ammonium Salt Polyurethanes

**DOI:** 10.1038/srep32140

**Published:** 2016-08-26

**Authors:** Wei He, Yi Zhang, Jiehua Li, Yunlong Gao, Feng Luo, Hong Tan, Kunjie Wang, Qiang Fu

**Affiliations:** 1 College of Polymer Science and Engineering, State Key Laboratory of Polymer Materials Engineering, Sichuan University, Chengdu 610065, China.; 2 High and New Technology Research Center of Henan Academy of Sciences, Zhengzhou 450002, China; 3 Department of Urology Surgery, West China Hospital, Sichuan University, Chengdu, 610041, China

## Abstract

Contact-active antibacterial surfaces play a vital role in preventing bacterial contamination of artificial surfaces. In the past, numerous researches have been focused on antibacterial surfaces comprising of antifouling upper-layer and antibacterial sub-layer. In this work, we demonstrate a reversed surface structure which integrate antibacterial upper-layer and antifouling sub-layer. These surfaces are prepared by simply casting gemini quaternary ammonium salt waterborne polyurethanes (GWPU) and their blends. Due to the high interfacial energy of gemini quaternary ammonium salt (GQAS), chain segments containing GQAS can accumulate at polymer/air interface to form an antibacterial upper-layer spontaneously during the film formation. Meanwhile, the soft segments composed of polyethylene glycol (PEG) formed the antifouling sub-layer. Our findings indicate that the combination of antibacterial upper-layer and antifouling sub-layer endow these surfaces strong, long-lasting antifouling and contact-active antibacterial properties, with a more than 99.99% killing efficiency against both gram-positive and gram-negative bacteria attached to them.

Microbial contamination occurring on artificial surfaces invariably leads to the formation of biofilms which have been associated with a variety of multi-resistant bacterial strains and resilient infections^1^,^2^. Moreover, biofilms may also cause material damage due to the secretion of embedded cells can degrade most man-made materials^3^,^4^. These hazards have threaten human health and caused functional failure in the applications of medical implants and devices^5^,^6^,^7^,^8^, water purification systems^9^,^10^, and ship hulls^11^,^12^, etc. To overcome these issues, various contact-active antibacterial materials derived from antibacterial polymers have been developed^3^,^13^,^14^. These materials are capable of perpetuating service life while avoiding the long-term cumulative toxicity originated from the release of biocides into the surrounding environment^3^,^15^,^16^,^17^. More importantly, contact-active antibacterial agents immobilized onto these material surfaces are less likely to induce resistant microbes^18^.

A significant issue with most current contact-active antibacterial surfaces is that they can easily be masked by biomolecules (such as proteins) or residues of dead cells, blocking further interactions with pathogens and triggering other undesired adverse effects^19^. The development of environmental friendly antibacterial materials that integrate contact-active biocidal and anti-fouling properties is therefore a promising approach to combat microbial contamination^20^,^21^. Recent approaches are based on a kind of surface combining biocidal sub-layer with antifouling upper-layer which is capable of repelling bacteria and killing anchored bacteria escaping from the antifouling upper-layer^22^,^23^. Such biocidal sub-layers within these surfaces are possibly blocked from further biocidal activity by dead adherent bacteria, which even leads to subsequent biofilms formation^23^. Evidently, the adhesion capacity of dead bacteria is much weaker than that of living bacteria, thus it is easier to repel dead bacteria from antifouling surfaces than living bacteria^1^. Therefore, an alternative to the method combining contact-active antimicrobial upper-layer and antifouling sub-layer will be more effective against bacterial contamination, of which surface is able to kill bacteria on contact first, and then eliminate the dead bacteria more effectively.

In our previous work, new antibacterial WPUs have been prepared using isophorone diisocyanate (IPDI), polytetramethylene glycol (PTMG), polyethylene glycol (PEG), a lysine-derivate of GQAS (N,N,N’,N’-tetramethyl-N,N’-bisdodecyl-2,6-bis(ammonium bromide)–L–lysine-(1′,3′- propylene diamide)–L-lysine, named EG12) and L-lysine via a simple polymerization process^24^. The structures of these WPUs are illustrated in Fig. 1. The samples are denoted as GWPU20, GWPU30, GWPU50, GWPU70 and GWPU100, of which the theoretical molar fraction of EG12 in chain extenders is 20, 30, 50, 70, and 100%, respectively. Also, polyurethane without EG12 as a blank is denoted as GWPU0 ((Supplementary Table S1). The chain extender EG12 applied in these WPUs possess much stronger surface activity and permanent excellent antibacterial activity than the normal single-chain quaternary ammonium salts^25^,^26^,^27^,^28^. More importantly, such antibacterial surfactant can accumulate at polymer/air interface to form antibacterial brushes because of the high interfacial energy^29^,^30^. Therefore, we demonstrate here novel contact-active antibacterial and antifouling surfaces constructed from these antibacterial GWPUs. These surfaces are comprised of GQAS antibacterial brushes and an antifouling sub-layer (Fig. 1). The antifouling sub-layer consisted of PEG and carboxyl anions of L-lysine could remove proteins and the residue from dead bacteria cells.

## Results and Discussion

### Surface characterization of GWPU films

For contact-active antibacterial materials, in general, high concentrations of antibacterial agents on their surfaces are desirable. Taking full advantage of high interfacial energy of GQAS, a series of special GWPU films containing antibacterial brushes over an antifouling layer are prepared by simply casting GWPU emulsions on the bottoms of siliconized culture dishes, followed by air-drying at room temperature. As a result, the GQAS in GWPUs could spontaneously migrate and aggregate onto the surfaces of the films during forming process. Therefore, the water contact angles (WCAs) of GWPUs are in the range of 59–64° at the beginning of contact, and rapidly decrease with contact time, falling to around 9–15° after 20 s (Fig. 2a, Supplementary Fig. S1). In contrast, the WCAs of the control (GWPU0) change from 80° to 58° during the observation period (Fig. 2a, Supplementary Fig. S1). Water could spread out on the surfaces of these GWPU films, indicating that the hydrophilicity of GWPU surfaces is significantly increased by the incorporation of GQAS^31^,^32^. These phenomena are mainly attributed to cationic GQAS migrating to the uppermost polyurethane surfaces^31^.

Comparing the attenuated total reflectance Fourier transform infrared spectroscopy (ATR-FTIR) spectra representing the surface character of these GWPUs with the transmission Fourier transform infrared (FTIR) spectra representing the bulk character, the adsorption intensities of peaks around 1650 cm^−1^ attributed to carbonyls in urea groups in the ATR-FTIR spectra (Fig. 2c, Supplementary Fig. S2b) are much stronger than those in transmission FTIR spectra (Fig. 2b, Supplementary Fig. S2a), and are enhanced as the GQAS concentration in these polyurethanes increased (Fig. 2c).

**Figure 1:**
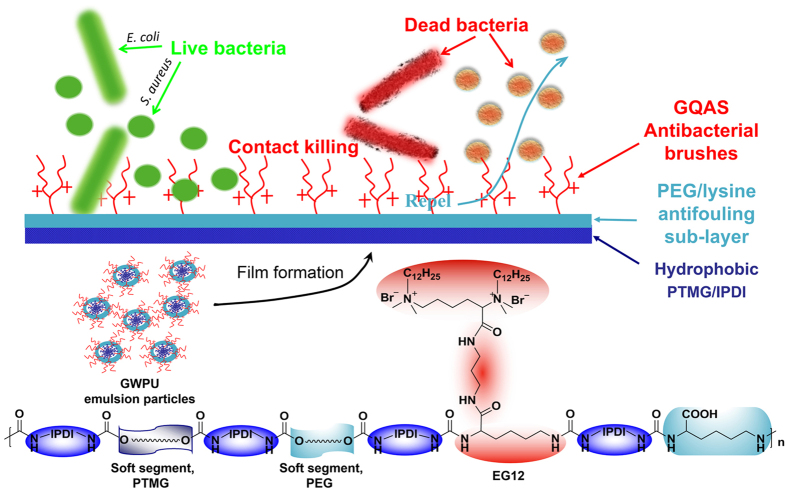
The schematic of antibacterial and antifouling gemini quaternary ammonium salt WPU (GWPU) films and the corresponding chemical structure.

**Figure 2:**
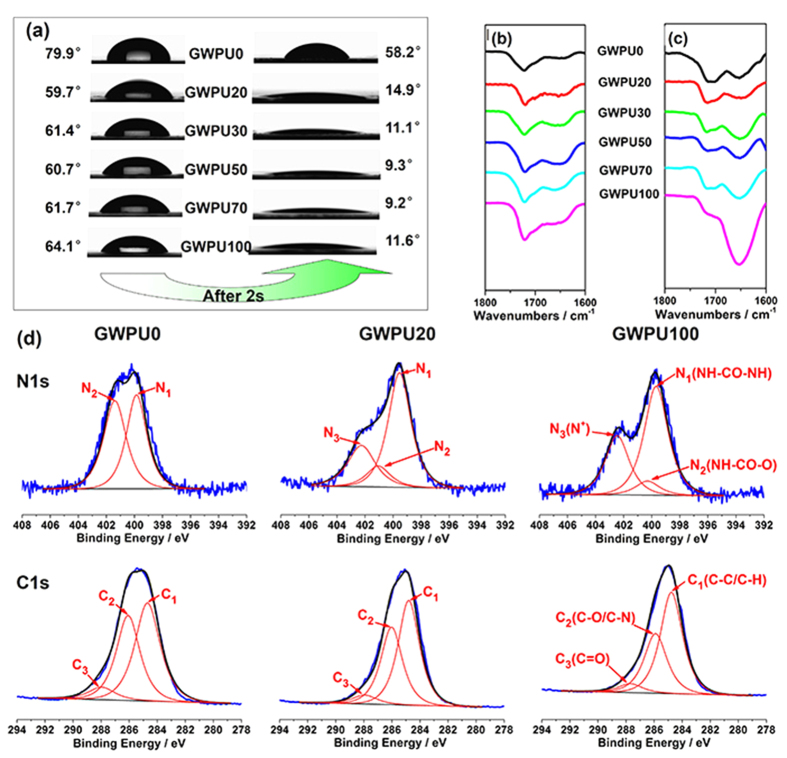
The surface structures of GWPU films. (**a**) WCAs of GWPU films. (**b**) The transmission FTIR spectra of GWPU films. (**c**) ATR-FTIR spectra of GWPU films. (**d**) High resolution N1s and C1s spectra of XPS at 30° take-off angle. For more details, see Supplementary Fig. S3.

To further verify and quantify the GQAS accumulating on the surfaces of the GWPU films, the atomic percentages of carbon, oxygen, and nitrogen on these surfaces are measured by X-ray Photoelectron Spectroscopy (XPS) (Fig. 2d, Supplementary Fig. S3, and Table S2). The atomic percentages of positive nitrogen atoms (N^+^) are taken to represent the amount of GQAS on the GWPU surfaces. The N1s peaks at around 402.5 eV originating from the GQAS nitrogen (N^+^) can be detected in GWPU20 and GWPU100 (Fig. 2d)^33^. The atomic percentages of N^+^ on these surfaces are 6.08 and 2.25 times higher than those in their corresponding bulks, which are up to 2.19% and 3.10%, respectively (Supplementary Table S2), again, suggesting that GQAS surfactants can accumulate at the film/air interfaces of GWPU films owing to their high interfacial energy^34^. These surfaces therefore have excellent antibacterial activity, the details will be discussed in a later section. Additionally, the atomic percentage of oxygen on the surface of GWPU0 films is slightly higher than in the bulks owing to the migration of PEG segments to the surfaces (Supplementary Table S2)^35^,^36^. For GWPU20 and GWPU100 samples, however, the reverse is true, probably because their uppermost surfaces are covered by GQAS (Supplementary Table S2). These results confirm that the surfaces of the GWPU films are formed by an upper GQAS antibacterial brush layer and an antifouling PEG and carboxyl anion sub-layer, as depicted in Fig. 1.

### Antibacterial properties of GWPU film surfaces

**Figure 3:**
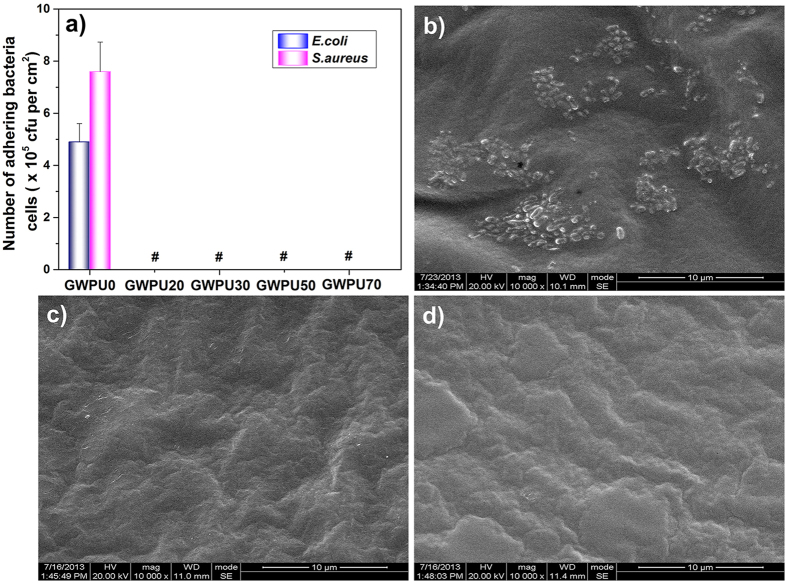
Antifouling and antimicrobial performances of GWPU films. (**a**) The number of living bacteria cells attaching on GWPU surfaces. # no bacteria cell was observed. (**b**) SEM image of GWPU0 film, (**c**) SEM image of GWPU20 film, (**d**) SEM image of GWPU20-7d film. Scale bars in (**b–d**) represent 10 μm.

To evaluate antibacterial and antifouling activities of these surfaces, these films are subjected to a measurement conducted by the shake-flask method^37^,^38^,^39^. We cannot detect any living *Escherichia coli* (*E. coli*) or *Staphylococcus aureus* (*S. aureus*) cells attaching to GWPU films, even those formed by GWPU20, the one with the lowest GQAS content (Fig. 3a, Supplementary Table S3). In contrast, 4.9 × 10^5^ CFU/ml living *E. coli* cells and 7.6 × 10^5^ CFU/ml living *S. aureus* cells are found attaching to the surface of GWPU0 films (Supplementary Table S3). This is corroborated by scanning electron microscope (SEM) images. GWPU0 films are covered by *E. coli* (Fig. 3b) while no bacteria cells or their residues stay on GWPU20 films (Fig. 3c), indicating that antifouling surfaces can merely restrain bacteria residue adhesion, instead of living bacteria. The long-term antibacterial and antifouling activities of GWPU20 films are also tested, with the results showing that the antibacterial and antifouling effects are not diminished, even after washing in water (110 rpm, 37 °C) for 7 days (Fig. 3d, Supplementary Fig. S4 and Table S3). These results demonstrate that all the GQAS-containing WPU films show excellent antibacterial and antifouling properties.

### Surface structure and contact-active antibacterial properties of GWPU blend films

**Figure 4:**
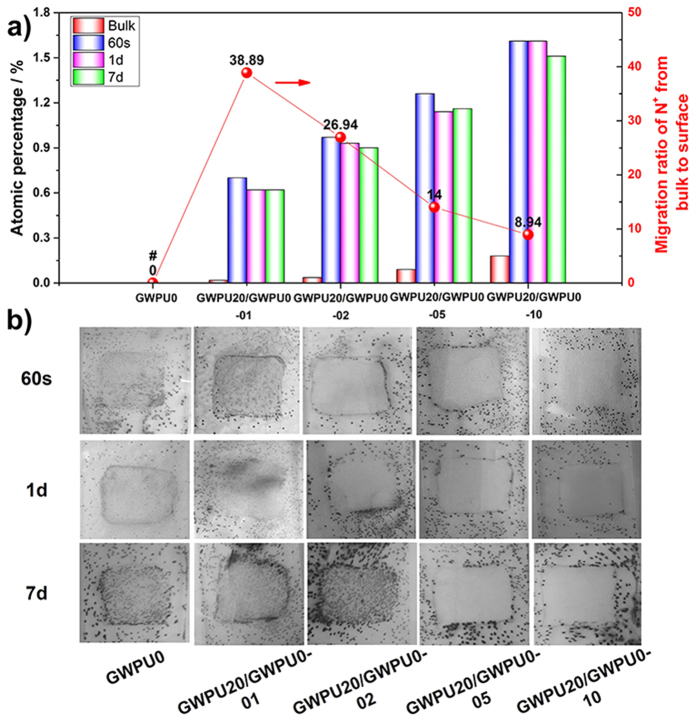
Surface structure and contact-active antibacterial activity of GWPU blends films. (**a**) Atomic percentages of N^+^ on the surface of the GWPU blend films (after 60 s, 1 day, and 7 days of washing in water, respectively) and migration ratio of N^+^ from bulk to surface of GWPU blend films obtained from XPS spectra. # no N^+^ was detected in GWPU0. (**b**) Photographs of GWPU blend films coated on glass slides with the size of 1.5 cm × 1.5 cm (after 60 s, 1 day and 7 days of washing in water, respectively) and sprayed with *S. aureus* aqueous suspensions (10^6^ CFU/ml) in phosphate buffer saline (PBS), air dried for 10 min, incubated with in a nutrient broth (0.8% agar) medium at 37 °C for 24 h, stained with 3 mL 5% 2, 3, 5-triphenyltetrazolium chloride (TTC). Each black dot corresponds to a bacterial colony grown from a single surviving bacteria cell.

To demonstrate that blending gemini-containing WPU with GWPU0 can also form such surfaces with an upper GQAS antibacterial brush layer and an antifouling sub-layer, a series of GWPU blends with different GQAS content (GWPU20/GWPU0-01, 02, 05, and 10, of which the theoretical molar fraction of GQAS in GWPU blends is 1, 2, 5 and 10%, respectively) are prepared. Even though the GQAS content of these blend films is dramatically reduced, their surfaces retained good hydrophilicity (Supplementary Fig. S1), because the GQAS moieties can readily migrate to the surfaces of blend films with a higher migration ratio (8.94–38.89) than that of the GWPU series (2.25–6.08). Besides, XPS analysis demonstrates that the atomic percentage of N^+^ on the surface is merely reduced a little, even in the blends films washed in water for 7 days (Fig. 4a, Supplementary Fig. S3, and Table S2).

Then, we employ a glass slide spreading method to investigate the contact-active antibacterial activity of the films made from these blends^12^, using GWPU0 as a negative control. No inhibition zones are observed, and their contact-active antibacterial activity enhance with increasing GWPU20 content (Fig. 4b). As a result, no *S. aureus* colonies are found on the films, including those which have been washed for 7 days in water prior to testing (Fig. 4b). The results suggest that effective contact-active antimicrobial activity against *S. aureus* requires a minimal concentration of antibacterial GQAS moieties in their bulks (0.99%, Supplementary Table S3), or a minimal N^+^ atomic percentage on the surfaces (0.93%, Fig. 4a, Supplementary Table S2).

**Figure 5:**
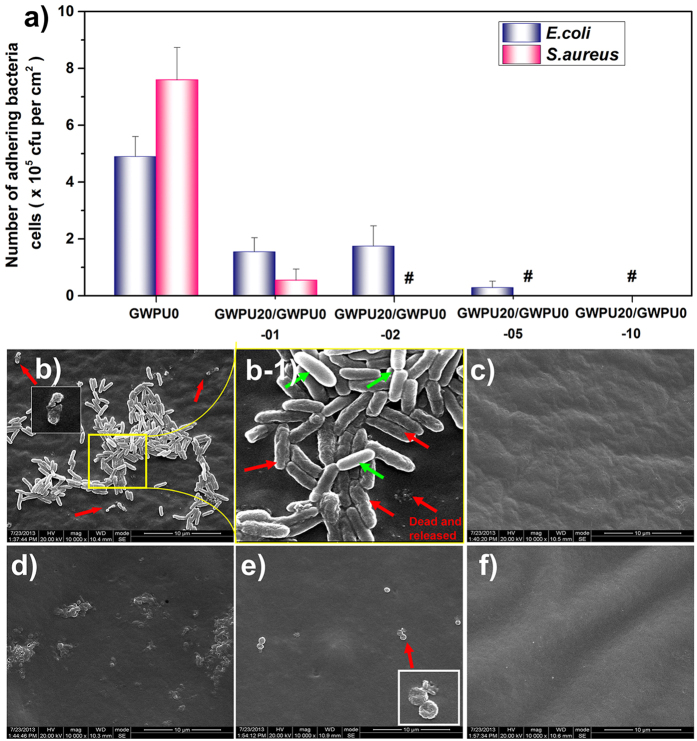
Antibacterial and antifouling performances of GWPU blends films. (**a**) Amounts of living bacteria cells attached on GWPU blend films. # no living bacteria cells were observed. Morphology of *E. coli* (**b,c**) and *S. aureus* (**d–f**) attached to GWPU blend films. (**b**) GWPU20/GWPU0-05, (b-1) The magnified image of (**b**), (**c**) GWPU20/GWPU0-10. (**d**) GWPU0, (**e**) GWPU20/GWPU0-01, (**f**) GWPU20/GWPU0-05. Red arrows indicate distortions and wrinkles in the membrane of dead bacteria after contact with the film; green arrows indicate living bacteria. Scale bars in b-f represent 10 μm.

To further evaluate the antibacterial and antifouling properties of blend films, the shake-flask method is employed. The GWPU20/GWPU0-01 surface containing 0.5 wt% EG12 could reduce surface-attached *E. coli* by 68.4%, and surface-attached *S. aureus* by 92.8% compared with GWPU0 film surfaces, and GWPU20/GWPU0-05 films (EG12: 2.48 wt%) exhibited a reduction of more than 99.99% in surface-attached *S. aureus*, and of 94.1% in surface-attached *E. coli* (Fig. 5a, Supplementary Table S3). As the EG12 content increase to 4.96 wt% (GWPU20/GWPU0-10), all surface-attached *E. coli* and *S. aureus* are killed (Fig. 5a, Supplementary Table S3). The result shows that the films containing more than 4.96 wt% EG12 have antifouling and antibacterial activity against both gram-positive and gram-negative bacteria. To demonstrate the mechanism through which attached bacteria are killed and their residues are dispersed, we use SEM to monitor morphological changes of the bacteria attaching to the films after 2 days of culture (Fig. 5b,f). The outcome of contact killing and shedding of attached *E. coli* can be observed in SEM images of GWPU20/GWPU0-05 (Fig. 5b). *E. coli* cell membranes first become distorted and wrinkled, and then broken, leading to cell death and the gradual shedding of residues from the film surface^40^. However, the *E. coli* adhering to other cells rather than directly attaching to films still retained their regular shape (Fig. 5b-1). Similar observations are made on *S. aureus* cells killed on the surface of a GWPU20/GWPU0-01 film (Fig. 5e). The contact-active antibacterial and antifouling mechanisms can thus be summarized as follows: both gram-positive and gram-negative bacterial cells contain a net negative charged outer envelope^41^. The main strategy for designing cationic GWPU has been determined by this common structural features. Cationic gemini ammonium salts can interact with the negative charged cell wall of gram-positive bacteria or the outer membrane of gram-negative bacteria or the cytoplasmic membrane of the both bacteria. Moreover, recent researches demonstrate that the positively charged quaternary ammonium salts on the polyurethane film surfaces first interact with the negatively charged phospholipid head groups of the bacteria cytoplasmic membrane, causing general perturbation of the lipid bilayer. The long hydrophobic alkyl chains then pierce the membranes of these surface-attached bacteria, forming holes that cause cytoplasm leakage, lysis, and death^42^,^43^. Because hydrophilic PEG chains combining with carboxyl anions exhibit a high resistance to protein adsorption^21^,^44^, the bacterial residues remaining on the film surface are shed and the contact-active antibacterial function restored (Fig. 5b,f).

## Conclusion

In summary, a series of novel surfaces containing contact-active antibacterial upper-layer and antifouling sub-layer are prepared by simply casting GWPUs and their blends, as in which antibacterial GQAS brushes are positioned above a PEG and carboxyl anion antifouling layer. At EG12 contents above 4.96 wt%, the GWPUs surfaces possesses strong, long-lasting antifouling and contact-active antibacterial activity, with a more than 99.99% killing efficiency against attached gram-positive and gram-negative bacteria. This novel surface structure may provide new insights for the better designing of contact-active antibacterial and antifouling surfaces. The GWPUs with non-fouling and antimicrobial properties could potentially be used in a wide range of biomedical and industrial applications.

## Materials and Methods

### Waterborne polyurethane preparation

Waterborne polyurethane and gemini quaternary ammonium salt waterborne polyurethane (GWPU) were synthesized as described in our previous report^24^. Briefly, GWPU emulsions are prepared using isophorone diisocyanate (IPDI), polytetramethylene glycol (PTMG), polyethylene glycol (PEG), a lysine-derivate of GQAS (N,N,N’,N’-tetramethyl-N,N’-bisdodecyl-2,6-bis(ammonium bromide)–L–lysine-(1′,3′- propylene diamide)–L-lysine, named EG12) and L-lysine via a simple polymerization process.

### Preparation of GWPU films

GWPU films were prepared through casting the GWPU emulsions on the surfaces of siliconized culture dishes and drying at room temperature for 2 days, then putting into an oven at 60 °C for 2 days, followed by 60 °C under vacuum for 2 days. The films were cut in sheets with 1 cm × 1 cm in size and approximately 0.5 mm thickness for physicochemical characterization. All GWPU films were immersed in water in a horizontal laboratory shaker (110 rpm, 37 °C) for 60 s, and then dried at 60 °C under vacuum for 2 days before testing, including antibacterial and antifouling activity, WCA, and ATR-FTIR measurements.

### Preparation of the waterborne GWPU blending films

Waterborne GWPU20 and GWPU0 were blended at the ratio of 1:19, 1:9, 1:3, 1:1, thus to obtain GWPU20/GWPU0-01, GWPU20/GWPU0-02, GWPU20/GWPU0-05, GWPU20/GWPU0-10 samples of these blends, respectively. These blend films were also prepared with a similar process described above.

### Samples for XPS analysis and contact-active antibacterial test

Samples were prepared through casting the emulsions (50 μl) onto cover glasses for XPS analysis and glass slides for contact-active antibacterial test (1.5 × 1.5 cm^2^) and drying. Then these samples were immersed in water at 37 °C for 60 s, 1 day or 7 days, respectively. The water was replaced every 12 hours in the first day, then every 2 days in the following time. Samples were taken out at the set time and dried in vacuum oven.

### Water contact angle (WCA) measurement

The surface hydrophilicity of various multi-block GWPUs is determined by contact angle measurement. Water contact angles were obtained on a Drop Shape Analysis System DSA 100 (Krüss, Hamburg, Germany) and 3 μL of distilled water at room temperature. The results were the mean values of three replicates.

### Fourier transform infrared (FTIR) spectra

Attenuated total reflectance-Fourier transform infrared spectroscopy (ATR-FTIR) was recorded on a Nicolet 6700 spectrometer (Thermo Electron Corporation, USA) between 4000 and 600 cm^−1^, with a resolution of 4 cm^−1^. Each sample spectrum was obtained by averaging 32 scans.

### X-ray photoelectron spectroscopy

X-ray photoelectron spectroscopy (XPS) was determined by a Kratos XSAM-800 Spectrometer with a Mg KR. The X-ray gun was operated at 20 kV and 10 mA current with a take-off angle of 30°. The relative atomic percentage of each element on the GWPU films and blend films was calculated by the peak areas using atomic sensitivity factors specified for the XSAM-800. C1s, O1s and N1s spectra bands were deconvoluted into sub-peaks by processing with the XPSPEAK4.0 spectrometer software.

### Evaluation of antibacterial and antifouling activities of GWPU films

To eliminate the influences of water-soluble antibacterial moieties onto these films of GWPUs on evaluation of their antibacterial and antifouling activities, all the films were immersed in water in a horizontal laboratory shaker (110 rpm, 37 °C) for 1 day or 7 days to a constant weight before the antibacterial test (Supplementary Fig. S4). The antibacterial and antifouling activities of these films were assessed against both *E. coli* (ATCC 25922) and *S. aureus* (ATCC 6538) according to shaking flask methods^37^,^38^,^39^. Each GWPU film (1.0 × 1.0 cm^2^, 0.5 mm thickness) was placed in a well of 24-well plate and sterilized under UV overnight. *E. coli* and *S. aureus* strain cultures in the nutrient broth (NB) were grown overnight at 37 °C and diluted to 10^7^ CFU/ml. This bacteria strain culture (2 mL) was added into each well with one GWPU film. The 24-well plate was then placed in a constant temperature incubator at 37 °C, 110 rpm for 2 days. After that, the films were taken out and rinsed three times with sterile deionized water, and then placed into another tube into which 2 mL of sterile water was added. The bacteria were detached in an ultrasonic cleaner for 5 min and diluted serially to proper concentration then counted by the flat colony counting method^37^. Each dilution was plated in triplicate on a nutrient agar plate and incubated at 37 °C for 24 h. The number of CFU at each dilution rate was counted after incubation and the average CFU/ml was determined.

### Contact-active antibacterial activity

GWPU films were transferred on to glass slides for the contact-active antibacterial test. Glass slides and GWPU films on them were sterilized by UV-irradiation for 30min before contact-active antibacterial test. In the test, aqueous suspensions of bacteria were sprayed onto both glass slides and GWPU films on glass slides followed by air-drying for 10 min. Afterward, the inoculated slides were transferred into Petri dishes and then the semi-solid nutrient agar (0.8% agar in a nutrient broth, autoclaved at 121 °C for 25 min, and cooled to 40 °C) was poured into Petri dishes slowly to make sure that the spayed cells would not be washed off. Then Petri dishes were sealed and placed in the incubator at 37 °C for 24 h. This allows the initially sprayed cells on glass slides and GWPU films to grow while being in contact with the surface. Finally, CFUs were stained with 3 ml 5% TTC (0.5 mg/ml) for observation and counting^43^.

### The antibacterial and antifouling activity of the blend GWPU films

As aforementioned in the part of evaluation of antibacterial and antifouling activities of GWPU films, the blend films were firstly washed in water (110 rpm, 37 °C) for 1 day or 7 days, and then the antibacterial and antifouling test were conducted. After 2 days incubation (110 rpm, 37 °C), the films were taken out and rinsed three times with sterile deionized water. The bacteria adhered to these films were counted by the flat colony counting method. The remaining films were immediately fixed with glutaraldehyde solution (2.5%) for 4 hours, then dehydrated by adding a graded series of aqueous ethanol (20%, 30%, and 50%) and dried in vacuum freeze drier. The bacteria adhered to the films were observed with scanning electron microscopy (SEM, Inspect F, FEI Company) for morphology changes. The numbers of live bacteria adhered to these GWPU and their blends films surface, and the antibacterial ratio of these film are shown in Supplementary Table S3.

## Additional Information

**How to cite this article**: He, W. *et al*. A Novel Surface Structure Consisting of Contact-active Antibacterial Upper-layer and Antifouling Sub-layer Derived from Gemini Quaternary Ammonium Salt Polyurethanes. *Sci. Rep.* **6**, 32140; doi: 10.1038/srep32140 (2016).

## Supplementary Material

Supplementary Information
