# Supplementary material for: A Novel Surface Structure Consisting of Contact-active Antibacterial Upper-layer and Antifouling Sub-layer Derived from Gemini Quaternary Ammonium Salt Polyurethanes
Source: Sci Rep. 2016 Aug 26;6:32140. doi: 10.1038/srep32140 (PMC4999876; doi:10.1038/srep32140)
Supplement: Supplementary Information [file srep32140-s1.pdf]

Supplementary Information

A Novel Surface Structure Consisting of Contact-active Antibacterial Upper-layer and Antifouling Sub-layer Derived from Gemini Quaternary Ammonium Salt Polyurethanes

Wei He[†1], Yi Zhang[†2], Jiehua Li*[1], Yunlong Gao[1], Feng Luo[1], Hong Tan*[1], Kunjie Wang[3], and Qiang Fu[1]

[1] College of Polymer Science and Engineering, State Key Laboratory of Polymer Materials Engineering, Sichuan University, Chengdu 610065, China. E-mail: hongtan@scu.edu.cn, jiehua_li@scu.edu.cn

[2] High and New Technology Research Center of Henan Academy of Sciences, Zhengzhou 450002, China

[3] Department of Urology Surgery, West China Hospital, Sichuan University, Chengdu, 610041, China

† These authors contributed equally.

Materials:

Waterborne polyurethane and gemini quaternary ammonium salt waterborne polyurethane (GWPU) were synthesized as described in our previous report (Table S1).[R1] Tryptone was supplied by Oxoid Ltd, UK. Beef powder was purchased from Beijing Solarbio Science&Technology Co., Ltd., China. Sodium chloride (NaCl) was obtained from Chengdu Kelong Chemical Co., Ltd., China. Agar was obtained from Biosharp, Japan. 2,3,5-triphenyltetrazolium chloride (TTC) was supplied by Sanland-chem International Inc. *Staphylococcus aureus* (*S. aureus*, gram-positive, ATCC 6538) and *Escherichia coli* (*E. coli*, gram-negative, ATCC 25922) were used for antibacterial and antifouling tests.

Table S1. Theoretical composition of GWPU samples

Samples	Molar ratio of IPDI/PEG/PTMG/ chain extender	Molar ratio of EG12/chain extender (%)	EG12 (mol %)	EG12 (wt%)
GWPU0	3:0.25:0.75:1.7	0	0	0
GWPU20	3:0.25:0.75:1.7	20	5.96	9.91
GWPU30	3:0.25:0.75:1.7	30	8.95	14.16
GWPU50	3:0.25:0.75:1.7	50	14.9	21.81
GWPU70	3:0.25:0.75:1.7	70	20.9	28.90
GWPU100	3:0.25:0.75:1.7	100	29.8	37.44

Water contact angle (WCA) measurement:

The surface hydrophilicity of various multi-block GWPUs is determined by contact angle measurements, and the data are illustrated in Fig. S1. The surface of GWPU0 without GQAS is hydrophilic because the new hydrogen bonds between water molecules and PEG segments or carboxyl groups aggregated onto the surfaces as soon as contacting with water.[R2] The WCAs on of GWPU are much lower than those of GWPU0, indicating that the hydrophilicity of GWPU films surface are significantly enhanced with the incorporation of EG12. The decreased time-related water contact angle and good hydrophilicity are attributed to the cationic nature of gemini quaternary ammonium polyurethanes on their surfaces. Slightly

rebound of the WCAs of GWPU70 and GWPU100 are ascribed to the migration of more hydrophobic long alkyl chain to the surface and the lower degree of surface rearrangement upon water contact.[R3,4]

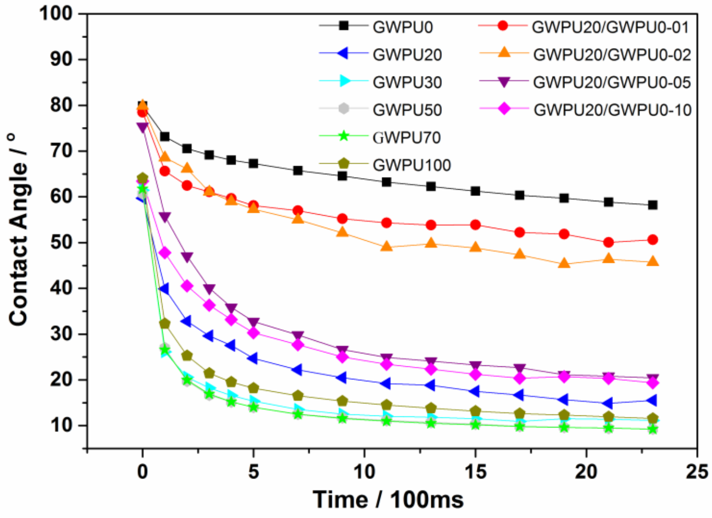

Figure S1. Time-related water contact angle of GWPU films surfaces

Fourier transform infrared (FTIR) spectra:

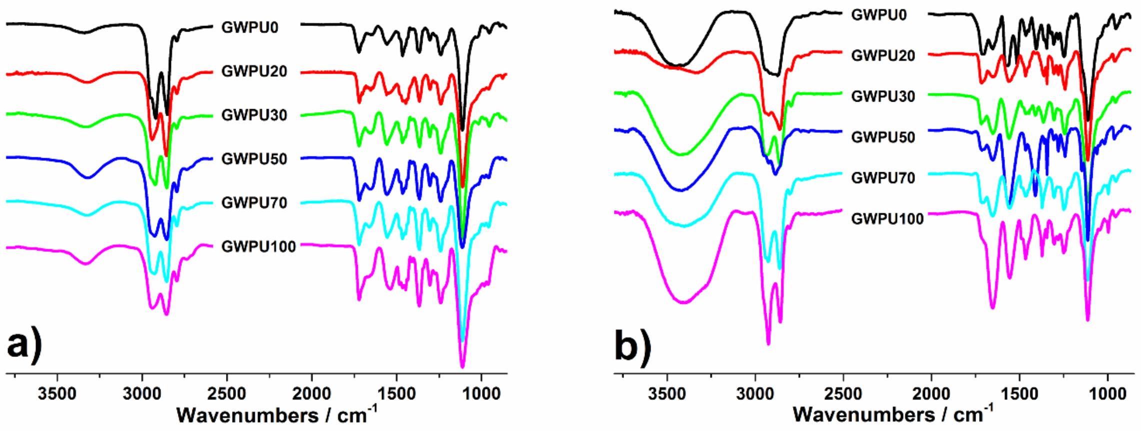

Figure S2. a): The transmission FTIR spectra of GWPU samples. b): The ATR- FTIR spectra of GWPU samples.

It can be concluded from the transmission FTIR spectra (Fig. S2a) that most N-H groups of GWPU are involved in hydrogen bonds and C–H deformation vibration in the methyl and methylene groups linked

with N positive ions are also observed at around 1,440 cm^{-1}.[1] Interestingly, the adsorption assigned to ν (C=O) of the carbonyl in carbamido groups of GWPU at around 1650 cm^{-1} in ATR-FTIR spectra (Fig. S2b) is much stronger than that of corresponding sample in transmission FTIR spectra (Fig. S2a), which is attributed to migration of most EG12 onto these surfaces, resulting in the aggregation of carbamido groups on the surfaces. In addition, the hydrophilic carboxyl group of lysine and PEG in GWPU0 are able to migrate onto the film surfaces, leading to certain growth of carbamido group in ATR-FTIR spectra of GWPU0 (Fig. S2b). These results indicate that these GWPU samples have been successfully prepared.

Samples for XPS analysis and contact-active antibacterial test:

Samples were prepared through casting the emulsions (50 μl) onto cover glasses for XPS analysis and glass slides for contact-active antibacterial test (1.5×1.5 cm^2) and drying. Then these samples were immersed in water at 37 ^{o}C for 60s, 1day and 7days, respectively. The water was replaced every 12 hours in the first day, then every 2 days in the following time. Samples were taken out at the set time and dried in vacuum oven.

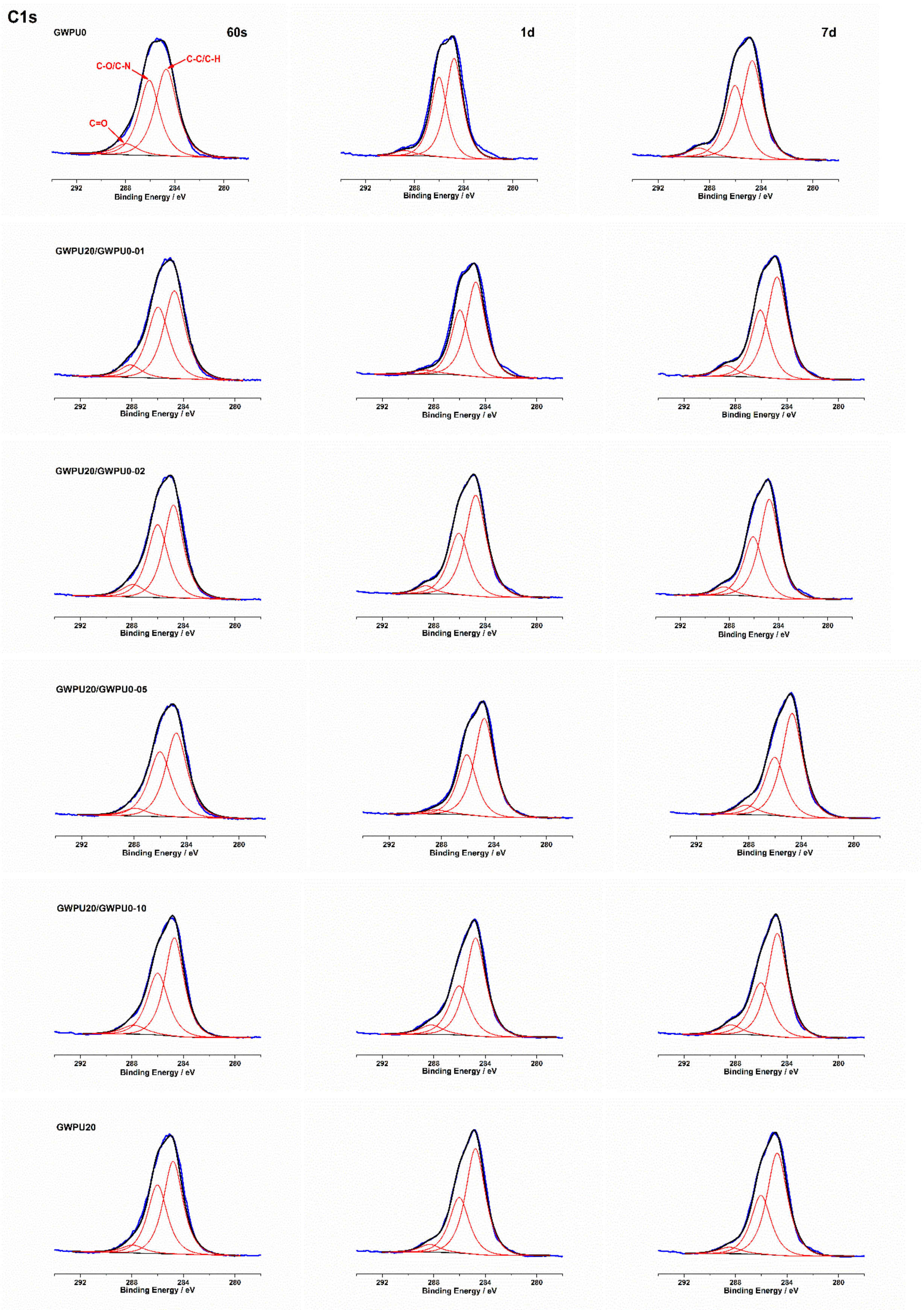
C1s
GWPU0
60s
1d
7d
C-O/C-N
C-C/C-H
C=O
GWPU20/GWPU0-01
GWPU20/GWPU0-02
GWPU20/GWPU0-05
GWPU20/GWPU0-10
GWPU20
292
288
284
280
Binding Energy / eV

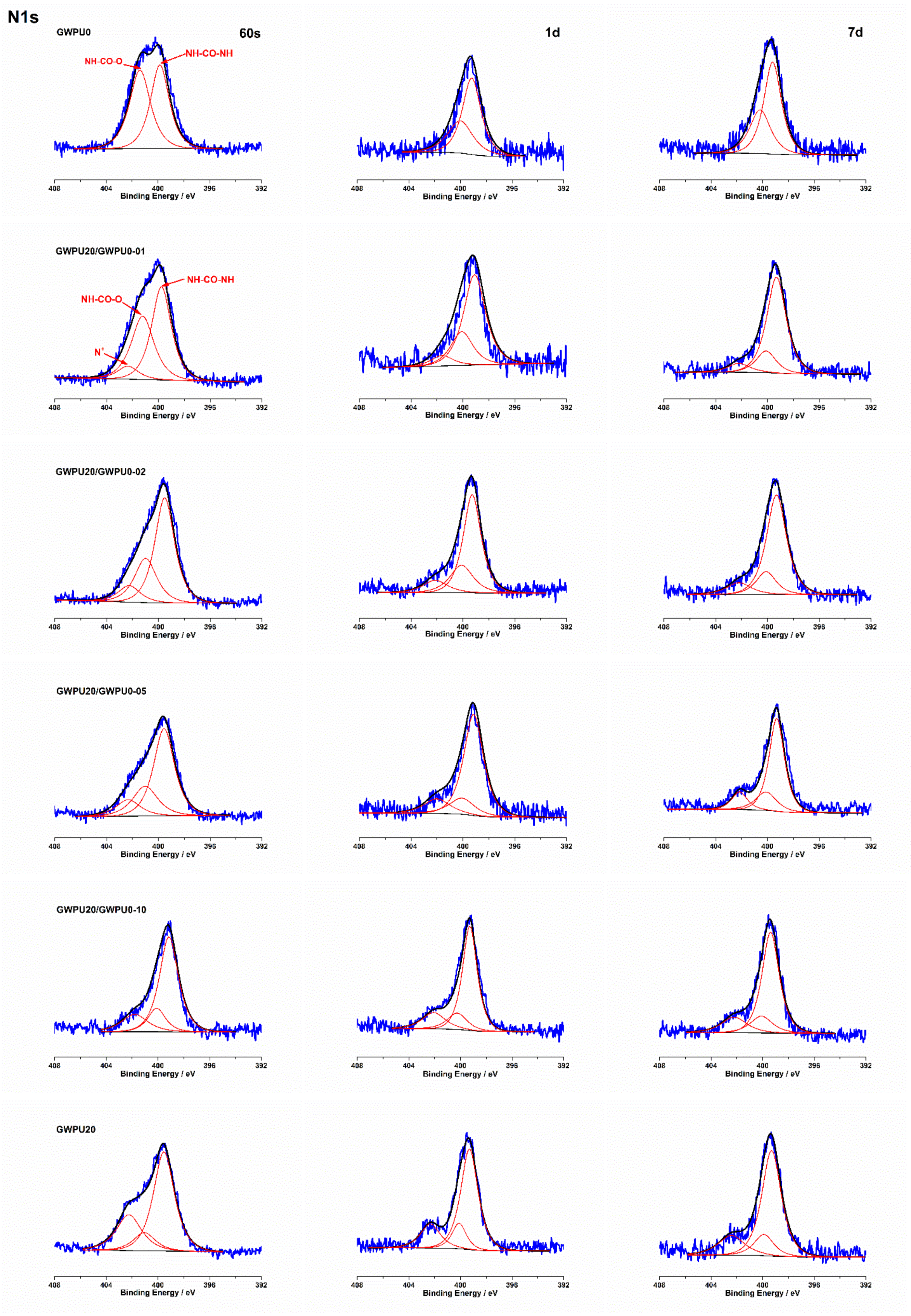

Figure S3. The C 1s and N 1s XPS spectra of GWPU films at 30° takeoff angle

Table S2. Atomic percentages on the surfaces of GWPU films from XPS spectra at 30 °takeoff angle.

	Samples	Carbon components			Nitrogen components			Surface atomic percentages			
		C=O (%)	C-C/C-H (%)	C-O/ C-N (%)	Urea N (%)	Urethane N(%)	N^+ (%)	O (%)	C (%)	N (%)	N^+ (%)
60s	GWPU0	7.26	50.09	42.65	46.95	53.05	0.00	22.86 (21.36[a])	69.54	7.60	0.00 (0.00[a])
	GWPU20/GWPU0-01	7.41	51.85	40.74	51.72	40.52	7.76	19.27	71.73	9.00	0.70 (0.018[a])
	GWPU20/GWPU0-02	7.84	50.75	41.42	60.71	28.57	10.71	18.12	72.83	9.06	0.97 (0.036[a])
	GWPU20/GWPU0-05	5.41	52.10	42.48	61.40	25.12	13.49	17.53	73.09	9.37	1.26 (0.09[a])
	GWPU20/GWPU0-10	5.56	55.62	38.09	66.67	16.67	16.67	17.73	72.63	9.64	1.61 (0.18[a])
	GWPU20	5.77	56.46	37.77	61.46	11.85	26.70	16.98 (19.80[a])	74.80	8.22	2.19 (0.36[a])
	GWPU100	5.61	58.84	35.55	59.81	7.7	32.49	8.89 (15.32[a])	81.58	9.53	3.10 (1.38[a])
1d	GWPU0	3.20	54.74	42.06	69.05	30.95	0.00	22.80	70.47	6.74	0.00
	GWPU20/GWPU0-01	2.78	60.49	36.73	66.33	26.02	7.65	20.52	71.44	8.04	0.62
	GWPU20/GWPU0-02	3.97	60.72	35.32	65.09	23.67	11.24	20.42	71.32	8.26	0.93
	GWPU20/GWPU0-05	2.89	61.33	35.79	71.79	14.65	13.55	20.12	71.49	8.38	1.14
	GWPU20/GWPU0-10	6.60	61.80	31.60	68.60	16.86	14.53	17.25	73.24	9.51	1.61
	GWPU20	4.78	62.50	32.72	67.48	14.11	18.40	16.89	74.27	8.83	1.63
7d	GWPU0	5.05	55.89	39.06	63.91	36.09	0.00	24.25	69.17	6.58	0.00
	GWPU20/GWPU0-01	6.21	58.76	35.03	72.75	19.63	7.62	22.31	69.59	8.11	0.62
	GWPU20/GWPU0-02	5.01	59.91	35.08	72.78	16.67	10.56	21.46	70.17	8.37	0.90
	GWPU20/GWPU0-05	5.75	60.41	33.83	68.57	18.29	13.14	17.23	73.91	8.86	1.16
	GWPU20/GWPU0-10	6.08	60.99	32.94	69.43	14.77	15.80	16.64	73.83	9.47	1.51
	GWPU20	4.22	61.33	34.44	68.28	14.20	17.52	14.73	76.75	8.51	1.49

[a]: The theoretical atomic percentages in bulk

It can be observed that peaks at around 284.8, 286.0 and 287.8 eV (Fig. S3 - C1s) are attributed to the functional groups of C-C/C-H, C-O/C-N, and C=O respectively, and peaks at around 399.9 and 400.2 eV (Fig. S3 - N1s) are corresponding to the nitrogen in NH-CO-NH and NH-CO-O groups respectively.[R5,6] The contents of C-C/C-H and C % on the upper surfaces of GWPU films escalate along with the increase of EG12 content (Table S2), even after 7d washing. The N1s peaks at binding energy around 402.4 eV originating from the quaternary ammonium nitrogen (N^+) are also enhanced with EG12 content increasing in all GWPU films (Fig.S3-N1s,Table S1). On the other hand, the concentrations of N in urethane groups, C=O and O % on GWPU surfaces decreased as EG12 increased. The possible reasons is that hydrophobic long alkyl chain of EG12 gradually moves to the water-air interface, leading to the aggregation of N^+ onto the film surfaces, simultaneously, the groups of C=O, N in urethane groups might be covered by long alkyl chain of EG12. These results show that surfactant EG12 could gradually move to the water-air interface and aggregating on the film surfaces in the film-forming process of waterborne polyurethane emulsions, as demonstrated by WCA, ATR-FTIR and XPS. Even though the contents of GQAS in these blends films are dramatically reduced, their surfaces retain good hydrophilicity, and antibacterial moieties GQAS could effortlessly migrate onto surfaces of these blends film with higher migration ratio (8.94-38.89). Alternatively, after 1 day and 7 days of washing in water, the atom percentage of quaternary ammonium nitrogen (N^+) on GWPU20 surface shows a significant reduction due to parts of GWPU dissolved in water. Fortunately, a minor reduction of N^+ is merely observed on the blend films surfaces because insoluble cross-linked GWPU0 could anchor soluble GWPU.

$$Migration\ ratio = \frac{N^+\ percentage\ on\ surface}{N^+\ percentage\ in\ bulk}$$

The antibacterial and antifouling activity of the GWPU blend films:

As shown in Table S3, it is more difficult to kill *E. coli* than *S. aureus* because it has an outer membrane. In our research, we found that the blend films containing more than 4.96 wt% EG12 have antifouling and antibacterial activity against both gram-positive and gram-negative bacteria. Thus, under the critical concentration (4.96 wt% EG12) for GWPU20/GWPU0-01 and GWPU20/GWPU0-02, it is not

stable for the blend films to kill *E. coli* or *S. aureus*, which possibly caused by heterogeneous charge density or charge distribution of these surfaces.

Table S3. The numbers of live bacteria adhered to these GWPU and their blends films surface, and the antibacterial ratio of these film.

Samples	Weight ratio of EG12/WPU (%)	*E.coli* (10^5 CFU/cm^2)	Killing efficiency of *E.coli* (%)	*S. aureus* (10^5 CFU/cm^2)	Killing efficiency of *S. aureus (%)*
GWPU20/GWPU0-01	0.5	1.55±0.49	68.36	0.55±0.39	92.76
GWPU20/GWPU0-02	0.99	1.75±0.71	64.28	0	99.99
GWPU20/GWPU0-05	2.48	0.29±0.23	94.08	0	99.99
GWPU20/GWPU0-10	4.96	0	99.99	0	99.99
GWPU0	0	4.90±0.71	0	7.60±1.13	0
GWPU20	9.91	0	99.99	0	99.99
GWPU20-7d	<9.91	0	99.99	0	99.99
GWPU30	14.16	0	99.99	0	99.99
GWPU50	21.81	0	99.99	0	99.99
GWPU70	28.90	0	99.99	0	99.99

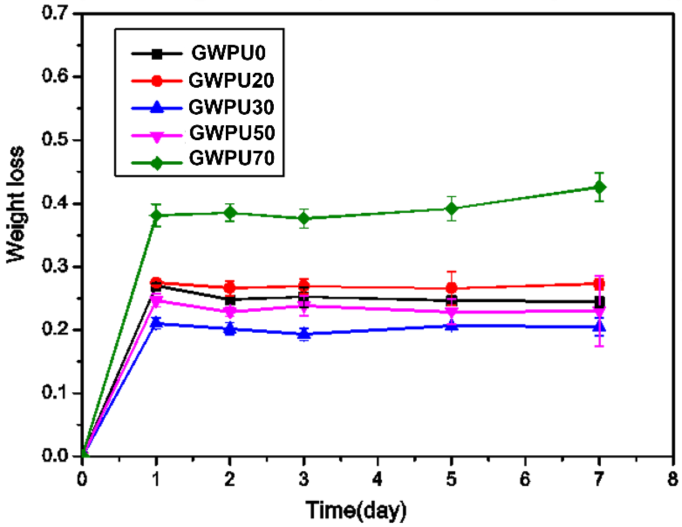

Figure S4. Weight loss of GWPU films after washing in water.

After 1 day washing, the weight of GWPU films had no big changes in the subsequent washing.

REFERENCES

R1 Zhang, Y. *et al.* Synthesis and antibacterial characterization of waterborne polyurethanes with gemini quaternary ammonium salt. *Sci. Bull.* **60**, 1114-1121 (2015).

R2 Song, N.-j., Jiang, X., Li, J.-h., Li, J.-s. & Fu, Q. The degradation and biocompatibility of waterborne biodegradable polyurethanes for tissue engineering. *Chin. J. Polym. Sci.* **31**, 1451-1462 (2013).

R3 Grapski, J. A. & Cooper, S. L. Synthesis and characterization of non-leaching biocidal polyurethanes. *Biomaterials* **22**, 2239-2246 (2001).

R4 Yung, L.-Y. L. & Cooper, S. L. Neutrophil adhesion on phosphorylcholine-containing polyurethanes. *Biomaterials* **19**, 31-40 (1998).

R5 Kocaefe, D. & Saha, S. Comparison of the protection effectiveness of acrylic polyurethane coatings containing bark extracts on three heat-treated North American wood species: Surface degradation. *Appl. Surf. Sci.* **258**, 5283-5290 (2012).

R6 Mishra, A. K., Chattopadhyay, D., Sreedhar, B. & Raju, K. FT-IR and XPS studies of polyurethane-urea-imide coatings. *Prog. Org. Coat.* **55**, 231-243 (2006).
